# Supplementary material for: Driver versus navigator causation in biology: the case of insulin and fasting glucose
Source: PeerJ. 2020 Dec 11;8:e10396. doi: 10.7717/peerj.10396 (PMC7735078; doi:10.7717/peerj.10396)
Supplement: Supplemental Information 1 [file peerj-08-10396-s001.docx]

**Supplementary information 1: Methods used for the systematic review**

**Four systematic reviews were performed in this study:**

1. Insulin receptor knockout

2. Insulin degrading enzyme

3. Insulin suppression by diazoxide

4. Insulin suppression by octreotide

**Keywords:** The keywords used for the four meta-analyses are given the following table

| **Sr. No.** | **Meta-analysis for** | **Keywords** |
| --- | --- | --- |
| 1. | Insulin receptor knockout | “insulin receptor knockout” |
| 2 | Insulin degrading enzyme | “insulin degrading enzyme” |
| 3. | Insulin suppression by diazoxide | “diazoxide and diabetes”; “insulin suppression” |
| 4. | Insulin suppression by octreotide | “octreotide and diabetes”; “insulin suppression |

**Data-bases:** We have used the PubMed/MEDLINE database (and not the data bases which report clinical trials data) since the experiments we were searching for are predominantly experiments in basic research in life sciences as opposed to clinical studies. Majority of the studies which were searching for were rodent studies and not human studies.

**Timeline for inclusion of papers in the search:** The first search was performed in August 2017 and the papers until 31^st^ July 2017 were included in the primary search.

**Inclusion and exclusion criteria:** Given in the tables 2,3 and 4 of the main paper

**Details of the papers:** Tables 2,3,4,5 below

**Methods of data extraction:** Data was extracted from the shortlisted papers using the software WebPlotDigitizer (Author: Ankit Rohatgi

Website: <https://automeris.io/WebPlotDigitizer>, Version: 4.1, January, 2018, E-Mail: [ankitrohatgi@hotmail.com](mailto:ankitrohatgi@hotmail.com), Location: Austin, Texas, USA)

**Principal summary measures:** The data extracted from each shortlisted paper was the difference of means of blood/plasma glucose levels between the ‘control’ and the ‘treated’ along with the 95% confidence intervals.

**Methods of handling data and combining results of studies:** These differences in the means between the control and treated from all the respective shortlisted papers were compiled. These differences were compared across different timepoints using the non-parametric chi-square test.

**Details of the papers shortlisted for the four meta-analyses:**

**Table 2:** Details of the 16 papers used in the Insulin Receptor Knock-Out (IRKO) analysis. All of these studies were carried out on rodent models.

| **Sr. No.** | **Reference** | **Type of IRKO** | **Method used to make the knockout** | **Fasting duration before the GTT (Glucose tolerance test)** | **Glucose concentration/ mode of glucose infusion used in GTT** | **Sample size** |
| --- | --- | --- | --- | --- | --- | --- |
| 1 | Sakaguchi et al., 2017 | inducible-BATIRKO (brown adipose tissue IRKO) | Cre-loxP system | 6 hours | 2g/kg dextrose given orally | Control n=13, IRKO, n=12 |
| 2 | Softic et al., 2016 | FIRKO (Fat IRKO) (12 weeks old male mice) | Cre-loxP system | Overnight (ON) | Random fed | n=12 to 30 for each group |
|  |  | FIRKO (52 weeks old male mice) |  |  |  | n=5 to 6 for each group |
| 3 | Haas et al., 2012 | LIRKO (Liver IRKO) | Cre-loxP system | ON | 1g/kg dextrose i.p.(intraperitoneal) | n=3 to 5 for each group |
| 4 | Kawamori et al., 2009 | αIRKO (α-cell IRKO) (2, 5, 12-month-old mice) | Cre-loxP system | 16 hours | Random fed | n=6 to 8 for each group |
|  |  | αIRKO (2,5-month-old mice) |  | 16 hours | 1g/kg dextrose i.p. | n=3 to 12 for each group |
| 5 | Escribano et al., 2009 | inducible LIRKO | Cre-loxP system | 16 hours | 2g/kg dextrose i.p. | n=10 to 20 for each group |
| 6 | Ealey et al., 2008 | MIRKO (Muscle IRKO) | Cre-loxP system | ON | 2g/kg dextrose i.p. | n=7 to 13 for each group |
| 7 | Okada et al., 2007 | βIRKO (β-cell IRKO), LIRKO and βIRKO-LIRKO (4-5 weeks old male mice) | Cre-loxP system | ON | 2g/kg dextrose i.p. | n=8 for each group |
|  |  | βIRKO (20 weeks old, male mice; chow and HFD) |  |  |  | n=9 to 16 for each group |
| 8 | Cohen et al., 2004 | LIRKO (2-month-old mice) | Cre-loxP system | 16 hours | 2g/kg dextrose i.p. | n=17 for control n=25 for LIRKO |
| 9 | Otani, 2003 | βIRKO-Non-diabetic (ND) | Cre-loxP system | 4 hours | 2g/kg dextrose i.p. | n= 35 for control, n=28 for βIRKO(ND) |
|  |  | βIRKO-Diabetic (D) |  |  |  | n=10 for βIRKO(D) |
| 10 | Blüher et al., 2002 | FIRKO (2 month and 10-month-old mice) | Cre-loxP system | 16 hours | 2g/kg dextrose i.p. | n=8 for each group |
| 11 | Guerra et al., 2001 | BATIRKO (3,6 and 9-month-old male and female mice) | Cre-loxP system | ON | 2g/kg dextrose i.p. | n=10 to 20 for each group |
| 12 | Lauro et al., 1998 | Insulin receptor (Ins R) and Ins R K1030 mutatnt | Cre-loxP system, | ON | 2g/kg dextrose i.p. | n=8 for each group |
| 13 | Mauvais-Jarvis et al., 2000 | MIRKO, βIRKO and βIRKO-MIRKO (2 and 6-month-old mice | Cre-loxP system | ON | 2g/kg dextrose i.p. | n=28 to 32 for each group |
| 14 | Dodson Michael et al., 2000 | LIRKO (2 and 6-month-old mice) | Cre-loxP system | 16 hours | 2g/kg dextrose i.p. | n=8 for each group |
| 15 | Wojtaszewski et al., 1999 | MIRKO | Cre-loxP system | ON | 2g/kg dextrose i.p. | n= 7 to 8 for each group |
| 16 | Brüning et al., 1998 | MIRKO | Cre-loxP system | ON | 2g/kg dextrose i.p. | n=8 for each group |

**Table 3:** Details of the 6 papers used in the Insulin Degrading Enzyme (IDE) inhibition analysis. All studies were carried out on rodent models.

| **Sr. No.** | **Reference** | **Method used to inhibit IDE** | **Fasting duration before the GTT** | **Glucose concentration/ mode of glucose infusion used in GTT** | **Sample size** |
| --- | --- | --- | --- | --- | --- |
| 1 | Villa-Pérez et al., 2018 | Liver specific IDE knockout | 16 hours | 2g/kg dextrose given i.p. | n= 9 to 13 for each group |
| 2 | Deprez-Poulain et al., 2015 | Inhibition of catalytic site of IDE using the inhibitor BDM44768 | 6 hours | 1.5g/kg glucose for IPGTT and 2 or 3g/kg glucose for OGTT | n= 4 to 7 for each group |
| 3 | Durham et al., 2015 | Inhibition of IDE using an N-terminal exosite (NTE) | ON | 2g/kg dextrose given orally | n=6 for each group |
| 4 | Maianti et al., 2014 | Inhibition of IDE using a non-catalytic site binding inhibitor | 14 hours | 1.5g/kg glucose for IPGTT and 3g/kg glucose for OGTT | n=5 to 7 for each group |
| 5 | Abdul-Hay et al., 2011 | IDE-KO created by Cre-lox recombination | 6 to 9 hours | 1g/kg dextrose given i.p. | n=10 to 12 for each group |
| 6 | Farris et al., 2003 | IDE^-/-^ mice created by gene trapping method | ON | 2g/kg dextrose given i.p. | n=6 (IDE^-/-^) n=4 (Control) |

**Table 4:** Details of the 8 papers used in the Diazoxide (DZX) analysis.

| **Sr. No.** | **Study reference** | **Concentration of diazoxide used** | **Details of subjects/model** | **Fasting duration** | **GTT details** | **Sample size for GTT (placebo, treatment)** |
| --- | --- | --- | --- | --- | --- | --- |
| **Studies on human subjects** | | | | | | |
| 1 | Brauner et al., 2016 | 3.2 to 4.2 mg/kg/d for 6months | Children over the age of 6 with hyperinsulinemia and obesity | ON | 75g glucose given to patients orally | n=12 to 17 for each group |
| 2 | Ramanathan et al., 2011 | 6mg/kg diazoxide | Healthy, young adults | ON | Mixed meal | n=11 for each group |
| 3 | van Boekel et al., 2008 | 50mg t.i.d (thrice in a day) for 4 weeks and then dose increased till 300mg t.i.d, total duration: 6 months | Obese, men, age 30 to 50 years | ON | Standardized mixed meal | n=18 for each group |
| 4 | Due et al., 2007 | 2mg/kg/day DZX or placebo for 8 weeks | 35 Overweight and obese men, age 23-54 years | ON | 75g glucose in 300m water given orally | n=13 (DZX) and n=18 (placebo) |
| 5 | Schreuder et al., 2005 | 50/75/100 mg t.i.d for 6 days | Healthy obese and non-obese men, age 30-50 years | ON | Standardized mixed meal | n=5 (non-obese) and n=12 (obese) |
| 6 | Wigand & Blackard, 1979 | 5mg/kg/d, 7 days | Obese, non-diabetic subjects, age 18-33 | ON | 40g/m^2^ body surface area glucose given orally | n=10 |
| **Studies on rodent models** | | | | | | |
| 7 | Matsuda et al., 2002 | 30mg/kg/day for 6 weeks | Male Wistar rats, control and STZ induced diabetes | 12 hours | 2g/kg glucose i.p | n=7 for each group |
| 8 | Leahy et al., 1994 | 30mg/kg/day, twice a day, for 8-12 days | Male Sprague-Dawley rats, 3 groups-sham, Pancreatectomised rats treated with water, pancreatectomised rats treated DZX | ON | 3.5g/kg oral gavage | n=4 for each group |

**Table 5:** Details of the 10 papers used in the Octreotide (OCT) analysis. All the papers included studies on human subjects.

| **Sr. No.** | **Study reference** | **Concentration of octreotide used** | **Details of subjects/model** | **Fasting duration** | **GTT details** | **Sample size for GTT (placebo, treatment)** |
| --- | --- | --- | --- | --- | --- | --- |
| 1 | Madsen et al., 2011 | Somatostatin analog (SA) alone:  OCT 10-30mg/4weeks OR Lanreotide 80mg/4weeks  Co-treatment:  OCT 6.7-20mg/4weeks OR Lanreotide 24-60mg/4weeks AND  Pegvisomat 30-60mg/4weeks | 18 Acromegalic patients (age 54±3 years) | ON | 75g glucose given orally | n=6 SA only  n=12 SA+P |
| 2 | Breckenridge et al., 2007 | OCT (30ng/kg.min) with GH +  1. Saline (treatment for our purpose)  2. Insulin (control for our purpose) | 14 Healthy adults (BMI 23 ± 2.9; Age 29 ± 5 years) | ON | 22.5µmol/kg | n=8 male n=6 female |
| 3 | Ronchi et al., 2002 | 1. Lanreotide (Slow Release)-30mg im injection every 14 days for 19±16 months  2. OCT (Long Acting Release)-20mg im injection every 28 days for 21±10 months | 10 acromegalic patients (6 men and 4 women; age 46±16 years; BMI 29±5) | ON | not mentioned | n=6 male n=4 female |
| 4 | Parkinson et al., 2002 | 1. OCT (50µg sc t.i.d) for 7 days  2. Pegvisomant (20mg/day sc) for 7 days | 6 healthy, male volunteers (age 21-63 years), studied on 3 separate occasions | ON | 75g glucose given orally | n=6 male |
| 5 | Giustina et al., 1991 | T2D patients received either of the four treatments  a. Insulin 0.1U/kg  b. OCT 25µg  c. OCT 50µg  d. OCT 100µg with insulin | 8 overweight/obese T2D patients (age 53.4 ± 4.2 years) | ON | Mixed meal | n=8 (7 female and 1 male) |
| 6 | Candrina et al., 1988 | Type 2 diabetes patients received 0.5U/kg/day divided into 2 subcutaneous injections | 5 T2D patients (age 56 ± 4 years), duration of diabetes ranged from 13 to 25 years | ON | 300 kcal breakfast | n=5 (3 male and 2 female) |
| 7 | Williams et al., 1988 | Type 2 diabetes patients received 50µg OCT, thrice a day, subcutaneously, for 3 days | 7 T2D patients (age 51-73 years, mean 67 years), duration of diabetes ranged from (6 months to 4 years, mean 2.5 years) | ON | Standardised breakfast | n=7 (4 males and 3 females) |
| 8 | Johnston et al., 1986 | 50 µg OCT administered subcutaneously, twice a day in diabetic and nondiabetic patients | 6 normal and 5 type 2 diabetic subjects (age range not given) | ON | Standardised meals | n=6 (normal, male) n=5 (T2D) |
| 9 | Davies et al., 1986 | 50 µg OCT administered subcutaneously, twice a day in diabetic patients | 5 T2D patients (mean age 49 years; duration of diabetes ranged from 3 to 10 years, with a mean of 6 years) | ON | Standardised breakfast | n=5 (male, diabetic) |
| 10 | Williams et al., 1986 | 5-100µg OCT administered subcutaneously in T2D patients twice a day Concentrations used:  50 µg in normal  5,100 µg in T2D | 5 normal and 5 T2D patients (age 50 to 65 years | ON | Standardised breakfast | n=5 (normal) n=5 (T2D) |

**References**

Abdul-Hay, S. O., Kang, D., McBride, M., Li, L., Zhao, J., & Leissring, M. A. (2011). Deletion of insulin-degrading enzyme elicits antipodal, age-dependent effects on glucose and insulin tolerance. *PloS One*, *6*(6), e20818. https://doi.org/10.1371/journal.pone.0020818

Blüher, M., Michael, M. D., Peroni, O. D., Ueki, K., Carter, N., Kahn, B. B., & Kahn, C. R. (2002). Adipose tissue selective insulin receptor knockout protects against obesity and obesity-related glucose intolerance. *Developmental Cell*. https://doi.org/10.1016/S1534-5807(02)00199-5

Brauner, R., Serreau, R., Souberbielle, J. C., Pouillot, M., Grouazel, S., Recasens, C., Zerah, M., Sainte-Rose, C., & Treluyer, J. M. (2016). Diazoxide in children with obesity after hypothalamic-pituitary lesions: A randomized, placebo-controlled trial. *Journal of Clinical Endocrinology and Metabolism*, *101*(12), 4825–4833. https://doi.org/10.1210/jc.2016-2126

Breckenridge, S. M., Cooperberg, B. A., Arbelaez, A. M., Patterson, B. W., & Cryer, P. E. (2007). Glucagon, in concert with insulin, supports the postabsorptive plasma glucose concentration in humans. *Diabetes*, *56*(10), 2442–2448. https://doi.org/10.2337/db07-0751

Brüning, J. C., Michael, M. D., Winnay, J. N., Hayashi, T., Hörsch, D., Accili, D., Goodyear, L. J., & Kahn, C. R. (1998). A muscle-specific insulin receptor knockout exhibits features of the metabolic syndrome of NIDDM without altering glucose tolerance. *Molecular Cell*. https://doi.org/10.1016/S1097-2765(00)80155-0

Candrina, R., Gussago, A., & Giustina, G. (1988). Effect of a new long-acting somatostatin analogue (SMS 201–995) on glycemic and hormonal response to a mixed meal in acromegalic patients. *Journal of Endocrinological Investigation*, *11*(1), 21–26. https://doi.org/10.1007/BF03350089

Cohen, S. E., Tseng, Y.-H., Michael, M. D., & Kahn, C. R. (2004). Effects of insulin-sensitising agents in mice with hepatic insulin resistance. *Diabetologia*, *47*(3), 407–411. https://doi.org/10.1007/s00125-003-1320-4

Davies, R. R., Miller, M., Turner, S. J., Watson, M., McGill, A., Orskov, H., Alberti, K. G., & Johnston, D. G. (1986). Effects of somatostatin analogue SMS 201-995 in non-insulin-dependent diabetes. *Clinical Endocrinology*, *25*(6), 739–747.

Deprez-Poulain, R., Hennuyer, N., Bosc, D., Liang, W. G., Enée, E., Marechal, X., Charton, J., Totobenazara, J., Berte, G., Jahklal, J., Verdelet, T., Dumont, J., Dassonneville, S., Woitrain, E., Gauriot, M., Paquet, C., Duplan, I., Hermant, P., Cantrelle, F. X., … Deprez, B. (2015). Catalytic site inhibition of insulin-degrading enzyme by a small molecule induces glucose intolerance in mice. *Nature Communications*, *6*. https://doi.org/10.1038/ncomms9250

Dodson Michael, M., Kulkarni, R. N., Postic, C., Previs, S. F., Shulman, G. I., Magnuson, M. A., & Ronald Kahn, C. (2000). Loss of Insulin Signaling in Hepatocytes Leads to Severe Insulin Resistance and Progressive Hepatic Dysfunction. *Molecular Cell*, *6*, 87–97.

Due, A., Flint, A., Eriksen, G., Møller, B., Raben, A., Hansen, J. B., & Astrup, A. (2007). No effect of inhibition of insulin secretion by diazoxide on weight loss in hyperinsulinaemic obese subjects during an 8-week weight-loss diet. *Diabetes, Obesity and Metabolism*, *9*(4), 566–574. https://doi.org/10.1111/j.1463-1326.2006.00645.x

Durham, T. B., Toth, J. L., Klimkowski, V. J., Cao, J. X. C., Siesky, A. M., Alexander-Chacko, J., Wu, G. Y., Dixon, J. T., McGee, J. E., Wang, Y., Guo, S. Y., Cavitt, R. N., Schindler, J., Thibodeaux, S. J., Calvert, N. A., Coghlan, M. J., Sindelar, D. K., Christe, M., Kiselyov, V. V., … Sloop, K. W. (2015). Dual exosite-binding inhibitors of insulin-degrading enzyme challenge its role as the primary mediator of insulin clearance in vivo. *Journal of Biological Chemistry*, *290*(33), 20044–20059. https://doi.org/10.1074/jbc.M115.638205

Ealey, K. N., Lu, S., Lau, D., & Archer, M. C. (2008). Reduced susceptibility of muscle-specific insulin receptor knockout mice to colon carcinogenesis. *Am J Physiol Gastrointest Liver Physiol*, *294*(3), G679-86. https://doi.org/10.1152/ajpgi.00526.2007

Escribano, O., Gómez-Hernández, A., Díaz-Castroverde, S., Nevado, C., García, G., Otero, Y. F., Perdomo, L., Beneit, N., & Benito, M. (2015). Insulin receptor isoform A confers a higher proliferative capability to pancreatic beta cells enabling glucose availability and IGF-I signaling. *Molecular and Cellular Endocrinology*. https://doi.org/10.1016/j.mce.2015.03.008

Farris, W., Mansourian, S., Chang, Y., Lindsley, L., Eckman, E. A., Frosch, M. P., Eckman, C. B., Tanzi, R. E., Selkoe, D. J., & Guenette, S. (2003). Insulin-degrading enzyme regulates the levels of insulin, amyloid beta-protein, and the beta-amyloid precursor protein intracellular domain in vivo. *Proceedings of the National Academy of Sciences of the United States of America*, *100*(7), 4162–4167. https://doi.org/10.1073/pnas.0230450100

Giustina, A., Girelli, A., Buffoli, M. G., Cimino, A., Legati, F., Valentini, U., & Giustina, G. (1991). Low-dose octreotide is able to cause a maximal inhibition of the glycemic responses to a mixed meal in obese type 2 diabetic patients treated with insulin. *Diabetes Research and Clinical Practice*, *14*(1), 47–54. https://doi.org/10.1016/0168-8227(91)90052-F

Guerra, C., Navarro, P., Valverde, A. M., Arribas, M., Brüning, J., Kozak, L. P., Kahn, C. R., & Benito, M. (2001). Brown adipose tissue – specific insulin receptor knockout shows diabetic phenotype without insulin resistance. *The Journal of Clinical Investigation*, *108*(8), 1205–1213. https://doi.org/10.1172/JCI200113103.Introduction

Haas, J. T., Miao, J., Chanda, D., Wang, Y., Zhao, E., Haas, M. E., Hirschey, M., Vaitheesvaran, B., Farese, R. V., Kurland, I. J., Graham, M., Crooke, R., Foufelle, F., & Biddinger, S. B. (2012). Hepatic Insulin Signaling Is Required for Obesity-Dependent Expression of SREBP-1c mRNA but Not for Feeding-Dependent Expression. *Cell Metabolism*, *15*(6), 873–884. https://doi.org/10.1016/j.cmet.2012.05.002

Johnston, D. G., Davies, R. R., Alberti, K. G. M. M., Miller, M., Turner, S. J., Watson, M., & Orskov, H. (1986). Effects of SMS 201-995 on intermediary metabolism and endocrine status in normal and diabetic humans. *The American Journal of Medicine*, *81*(6 SUPPL. 2), 88–93. https://doi.org/10.1016/0002-9343(86)90589-9

Kawamori, D., Kurpad, A. J., Hu, J., Liew, C. W., Shih, J. L., Ford, E. L., Herrera, P. L., Polonsky, K. S., McGuinness, O. P., & Kulkarni, R. N. (2009). Insulin Signaling in α Cells Modulates Glucagon Secretion In Vivo. *Cell Metabolism*, *9*(4), 350–361. https://doi.org/10.1016/j.cmet.2009.02.007

Lauro, D., Kido, Y., Castle, a L., Zarnowski, M. J., Hayashi, H., Ebina, Y., & Accili, D. (1998). Impaired glucose tolerance in mice with a targeted impairment of insulin action in muscle and adipose tissue. *Nature Genetics*, *20*(3), 294–298. https://doi.org/10.1038/3112

Leahy, J. L., Bumbalo, L. M., & Chen, C. (1994). Diazoxide causes recovery of beta-cell glucose responsiveness in 90% pancreatectomized diabetic rats. *Diabetes*, *43*(2), 173–179. https://doi.org/10.1046/j.1464-5491.1999.00150.x

Madsen, M., Poulsen, P. L., Ørskov, H., Møller, N., & Jørgensen, J. O. L. (2011). Cotreatment with pegvisomant and a Somatostatin Analog (SA) in SA-responsive acromegalic patients. *Journal of Clinical Endocrinology and Metabolism*, *96*(8), 2405–2413. https://doi.org/10.1210/jc.2011-0654

Maianti, J. P., McFedries, A., Foda, Z. H., Kleiner, R. E., Du, X. Q., Leissring, M. A., Tang, W.-J., Charron, M. J., Seeliger, M. A., Saghatelian, A., & Liu, D. R. (2014). Anti-diabetic activity of insulin-degrading enzyme inhibitors mediated by multiple hormones. *Nature*, *511*(7507), 94–98. https://doi.org/10.1038/nature13297

Matsuda, M., Kawasaki, F., Mikami, Y., Takeuchi, Y., Saito, M., Eto, M., & Kaku, K. (2002). Rescue of beta-cell exhaustion by diazoxide after the development of diabetes mellitus in rats with streptozotocin-induced diabetes. *European Journal of Pharmacology*, *453*(1), 141–148. https://doi.org/10.1016/S0014-2999(02)02389-0

Mauvais-Jarvis, F., Virkamaki, A., Michael, M. D., Winnay, J. N., Zisman, A., Kulkarni, R. N., & Kahn, C. R. (2000). A model to explore the interaction between muscle insulin resistance and beta-cell dysfunction in the development of type 2 diabetes. *Diabetes*. https://doi.org/10.2337/diabetes.49.12.2126

Okada, T., Liew, C. W., Hu, J., Hinault, C., Michael, M. D., Krtzfeldt, J., Yin, C., Holzenberger, M., Stoffel, M., & Kulkarni, R. N. (2007). Insulin receptors in beta-cells are critical for islet compensatory growth response to insulin resistance. *Proceedings of the National Academy of Sciences of the United States of America*, *104*(21), 8977–8982. https://doi.org/10.1073/pnas.0608703104

Otani, K. (2003). Reduced  -cell mass and altered glucose sensing impair insulin-secretory function in  IRKO mice. *AJP: Endocrinology and Metabolism*. https://doi.org/10.1152/ajpendo.00533.2001

Parkinson, C., Drake, W. M., Roberts, M. E., Meeran, K., Besser, G. M., & Trainer, P. J. (2002). A comparison of the effects of pegvisomant and octreotide on glucose, insulin, gastrin, cholecystokinin, and pancreatic polypeptide responses to oral glucose and a standard mixed meal. *Journal of Clinical Endocrinology and Metabolism*, *87*(4), 1797–1804. https://doi.org/10.1210/jcem.87.4.8432

Ramanathan, R. P., Arbeláez, A. M., & Cryer, P. E. (2011). Partial inhibition of insulin secretion results in glucose intolerance but not hyperglucagonemia. *Diabetes*, *60*(4), 1324–1328. https://doi.org/10.2337/db10-1586

Ronchi, C., Epaminonda, P., Cappiello, V., Beck-Peccoz, P., & Arosio, M. (2002). Effects of two different somatostatin analogs on glucose tolerance in acromegaly. *Journal of Endocrinological Investigation*, *25*(6), 502–507. https://doi.org/10.1007/BF03345491

Sakaguchi, M., Fujisaka, S., Cai, W., Winnay, J. N., Konishi, M., O’Neill, B. T., Li, M., García-Martín, R., Takahashi, H., Hu, J., Kulkarni, R. N., & Kahn, C. R. (2017). Adipocyte Dynamics and Reversible Metabolic Syndrome in Mice with an Inducible Adipocyte-Specific Deletion of the Insulin Receptor. *Cell Metabolism*, *25*(2), 448–462. https://doi.org/10.1016/j.cmet.2016.12.008

Schreuder, T., Karreman, M., Rennings, A., Ruinemans-Koerts, J., Jansen, M., & de Boer, H. (2005). Diazoxide-mediated insulin suppression in obese men: a dose-response study. *Diabetes, Obesity & Metabolism*, *7*(3), 239–245. https://doi.org/10.1111/j.1463-1326.2004.00449.x

Softic, S., Boucher, J., Solheim, M. H., Fujisaka, S., Haering, M. F., Homan, E. P., Winnay, J., Perez-Atayde, A. R., & Kahn, C. R. (2016). Lipodystrophy due to adipose tissue-specific insulin receptor knockout results in progressive NAFLD. *Diabetes*, *65*(8), 2187–2200. https://doi.org/10.2337/db16-0213

van Boekel, G., Loves, S., van Sorge, A., Ruinemans-Koerts, J., Rijnders, T., & de Boer, H. (2008). Weight loss in obese men by caloric restriction and high-dose diazoxide-mediated insulin suppression. *Diabetes, Obesity and Metabolism*, *10*(12), 1195–1203. https://doi.org/10.1111/j.1463-1326.2008.00878.x

Villa-Pérez, P., Merino, B., Fernández-Díaz, C. M., Cidad, P., Lobatón, C. D., Moreno, A., Muturi, H. T., Ghadieh, H. E., Najjar, S. M., Leissring, M. A., Cózar-Castellano, I., & Perdomo, G. (2018). Liver-specific ablation of insulin-degrading enzyme causes hepatic insulin resistance and glucose intolerance, without affecting insulin clearance in mice. *Metabolism*, *88*, 1–11. https://doi.org/10.1016/J.METABOL.2018.08.001

Wigand, J. P., & Blackard, W. G. (1979). Downregulation of insulin receptors in obese man. *Diabetes*, *28*(4), 287–291. https://doi.org/10.2337/diab.28.4.287

Williams, G., Fuessl, H., Kraenzlin, M., & Bloom, S. R. (1986). Postprandial effects of SMS 201-995 on gut hormones and glucose tolerance. *Scandinavian Journal of Gastroenterology*, *21*(S119), 73–83. https://doi.org/10.3109/00365528609087434

Williams, G., Fuessl, H. S., Burrin, J. M., Chilvers, E., & Bloom, S. R. (1988). Postprandial glycaemic effects of a long-acting somatostatin analogue (octreotide) in non-insulin dependent diabetes mellitus. *Hormone and Metabolic Research*, *20*(3), 168–170. https://doi.org/10.1055/s-2007-1010784

Wojtaszewski, J. F. P., Higaki, Y., Hirshman, M. F., Michael, M. D., Dufresne, S. D., Kahn, C. R., & Goodyear, L. J. (1999). Exercise modulates postreceptor insulin signaling and glucose transport in muscle-specific insulin receptor knockout mice. *Journal of Clinical Investigation*. https://doi.org/10.1172/JCI7961
