# Supplementary material for: Driver versus navigator causation in biology: the case of insulin and fasting glucose
Source: PeerJ. 2020 Dec 11;8:e10396. doi: 10.7717/peerj.10396 (PMC7735078; doi:10.7717/peerj.10396)
Supplement: Supplemental Information 2 [file peerj-08-10396-s002.docx]

**Supplementary information 2**

**A generalized CSS model to make predictions testable in population data**

A number of models of glucose regulation exist in literature. We use a simple model assuming the following. The plasma glucose level *G* increases by two processes namely absorption from gut and glucose production by the liver. We assume the gut absorption *Gt* to be independent of standing plasma glucose as well as insulin, whereas liver glucose production has a maximum rate *L* which has two feedback inhibitors namely direct feedback inhibition by glucose and that by standing plasma insulin which depends upon the insulin sensitivity of liver. Glucose clearance has two mechanisms namely insulin independent and insulin dependent. The plasma insulin *I* is a balance between insulin release by pancreatic beta cells, the rate being a function of plasma glucose and a rate of insulin degradation which is directly proportional to standing plasma insulin level. We assume all relationships to be linear and use the model framework of Chawla et al 2018 (Chawla et al., 2018).

$$\frac{dG}{dt}=Gt+L-K_{1}.G-I_{SENS}.K_{2}.I$$

$$\frac{dI}{dt}=K_{3}.G-d.I$$

Where *K_1_* is a rate constant for glucose uptake by tissues as well as direct feedback inhibition of liver glucose production, *K_2_* a rate constant for insulin mediated inhibition of liver glucose production as well as insulin mediated glucose uptake, both of which are assumed to be a function of insulin sensitivity *I_SENS_* which is assumed to be unity normally and decreases with insulin resistance. *K_3_* is the rate constant for glucose stimulated insulin secretion and *d* the rate of insulin clearance.

We use simulations with normally distributed errors to study how the correlation between plasma glucose and insulin is affected by the parameters as well as by the standard deviation of errors. We use the errors additively or multiplicatively. For simulations using additive errors, we add normally distributed error terms *e_1_* and *e_2_* to both the equations.

$$\frac{dG}{dt}=L-K_{1}.G-I_{SENS}.K_{2}.I+e_{1}$$

$$\frac{dI}{dt}=K_{3}.G-d.I+e_{2}$$

For simulations using multiplicative error, we give normal distributions to *K_1_, K_2_, K_3_* and *I_SENS_*. Realistic ranges for the parameters are taken from Chawla et al 2018(Chawla et al., 2018).

Simulations show that in a additive error model, as long as the parameters of glucose insulin relationship are the same, the regression correlation parameters for glucose insulin relationship are not significantly different during fasting steady state (*Gt=0*) and at any time post meal (*Gt > 0*). The only difference is in the range of glucose and insulin distribution (figure 1)

**Figure 1:** The glucose insulin scatter in a fasting steady state (red squares) and in a post meal arbitrary but constant time interval (blue diamonds) in an additive error model. A sample result is shown in which *K_1_*=0.1, *K_2_*=0.9, *I_SENS_* is randomized between 0.1 and 1 and *K_3_*=0.015 and *d*=0.15. The error standard deviations are 15 and 1 respectively.

In simulations with multiplicative errors, the post meal glucose insulin correlation was always weaker than the fasting steady state correlation (figure 2). This is the likely result of the errors growing in proportion to larger values of glucose and insulin, and also due to an additional variable, gut absorption being incorporated in the model.

**Figure 2:** The glucose insulin scatter in a fasting steady state (red squares) and at a post meal arbitrary but constant time point (blue diamonds) in a multiplicative error model. A sample result is shown in which the mean (standard deviations) of the parameters were *K_1_*=0.1 (0.02), *K_2_*=0.9 (0.5), *I_SENS_* is randomized between 0.1 and 1 *K_3_*=0.0015 (0.0002) and *d* =0.15 (0.005). In all the simulations the correlation coefficient and regression slopes of the post meal scatters were less than or equal to the corresponding fasting parameters. This contrasts the epidemiological patterns in which the fasting correlations are substantially weaker than the post meal correlations (main text Table 7 figure 11).

The results were not sensitive to parameter changes as long as G and I were positive. We can confidently make a generalization that as long as the model parameters remain the same, the glucose insulin correlation in steady state is stronger or equal to the post meal correlation. Logically and intuitively sound, this generalization is unlikely to be specific to a particular form of equations based on the assumptions of the CSS class of models.

The simulation results contrast with real life data in which the steady state correlation and regression slope between glucose and insulin is observed to be substantially weaker than the post meal relations at any point in time. This indicates that the parameters of glucose insulin relationship in steady state are substantially different from the post meal parameters, or glucose insulin relationship in steady state is qualitatively different from that in the perturbed state.

**References**

Chawla, S., Pund, A., B., V., Kulkarni, S., Diwekar-Joshi, M., & Watve, M. (2018). Inferring causal pathways among three or more variables from steady-state correlations in a homeostatic system. *PLOS ONE*, *13*(10), e0204755. https://doi.org/10.1371/journal.pone.0204755
