# Supplementary material for: Driver versus navigator causation in biology: the case of insulin and fasting glucose
Source: PeerJ. 2020 Dec 11;8:e10396. doi: 10.7717/peerj.10396 (PMC7735078; doi:10.7717/peerj.10396)
Supplement: Supplemental Information 3 [file peerj-08-10396-s003.docx]

**Supplementary information 3: Additional information about the methods of the meta analyses**

**Systematic Review Registration and Rationale for the Meta-analyses:**

1. The fore meta-analyses were registered in PROSPERO.
2. We did not start the project with the intention of doing these systematic reviews. Our primary goal was to test the alternative evolutionary hypotheses for the origins of type 2 diabetes (Watve & Diwekar-Joshi, 2016). While pursuing this work we noticed certain basic anomalies in the classical theories of diabetes. At the stage when we thought of the question addresses in this paper, we had already scanned much of the literature used here.
3. To ensure that our review is unbiased and rigorous enough, we repeated the literature search following Prisma and PROSPERO registration. Therefore, much of the search for the meta analyses was done before the actual PROSPERO registration. The secondary screening, data extraction and the data analysis was done during the time required after the submission to the PROSPERO and the actual approval of the registration by them.
4. But we were glad to find after following proper procedures that our prior work met the standards of unbiased and rigorous review.
5. Details about the search strategies, quality assessment and data analysis for the four meta analyses are included in Supplementary information 1.

**Systematic Review/Meta-analyses:**

1. Manawa Diwekar-Joshi and Milind Watve decided the search strategy and the actual searches and screening based on the strategy was done by Manawa Diwekar-Joshi. Data analysis was done by both the authors.
2. Disagreements were dissolved by discussion and input from other lab members.
3. The referees for the manuscript were other post-doc and graduate students of the Watve Lab.

**References**

Watve, M., & Diwekar-Joshi, M. (2016). What to expect from an evolutionary hypothesis for a human disease: The case of type 2 diabetes. *HOMO*, *67*(5), 349–368. https://doi.org/10.1016/j.jchb.2016.07.001
