# Supplementary material for: Driver versus navigator causation in biology: the case of insulin and fasting glucose
Source: PeerJ. 2020 Dec 11;8:e10396. doi: 10.7717/peerj.10396 (PMC7735078; doi:10.7717/peerj.10396)
Supplement: Supplemental Information 7 — Moher D, Liberati A, Tetzlaff J, Altman DG, The PRISMA Group (2009). Preferred Reporting Items for Systematic Reviews and Meta-Analyses: The PRISMA Statement. PLoS Med 6(7): e1000097. doi:10.1371/journal.pmed1000097 [file peerj-08-10396-s007.pdf]

PRISMA 2009 Flow Diagram- Octreotide (DZX) meta-analysis

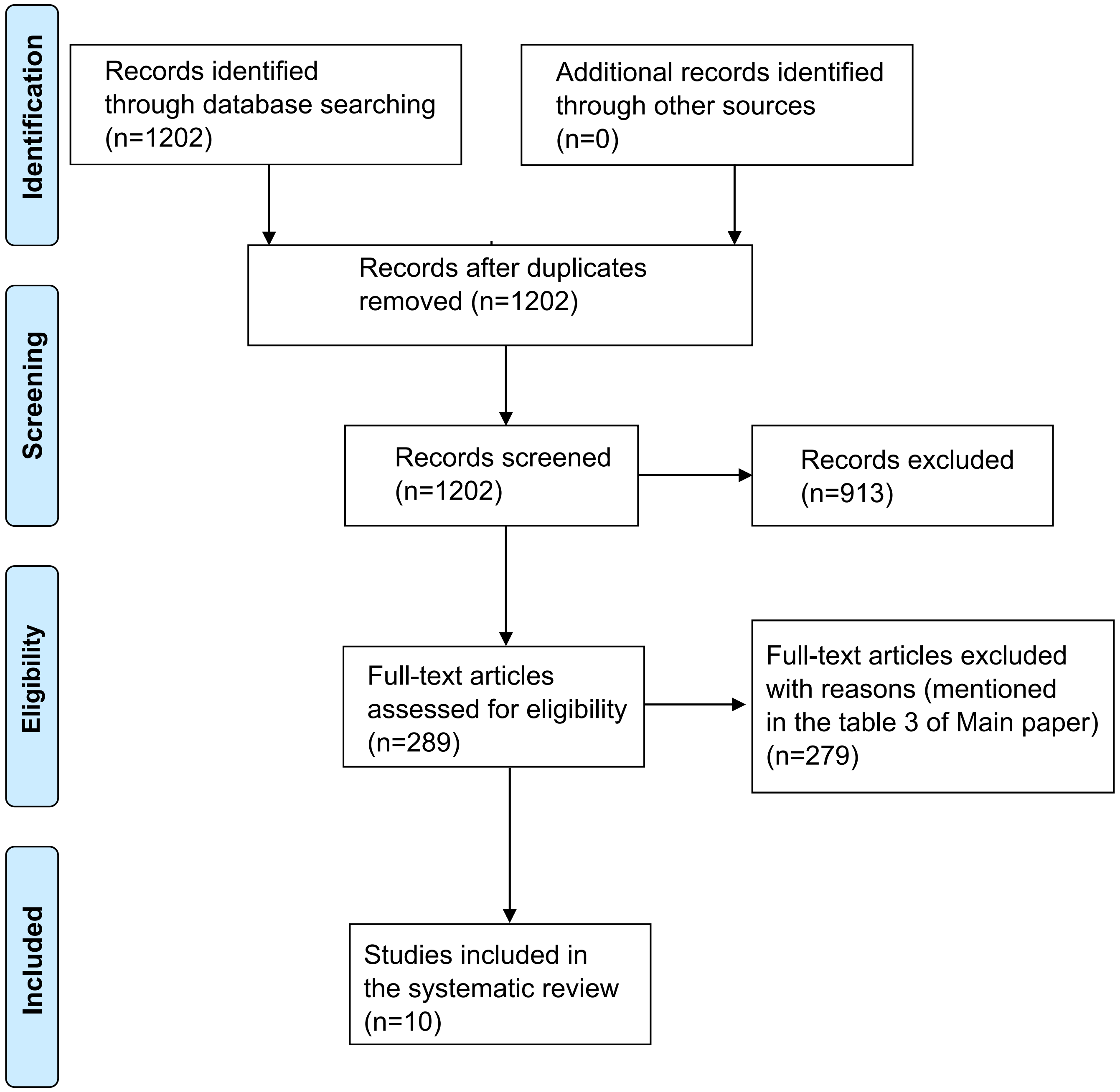

From: Moher D, Liberati A, Tetzlaff J, Altman DG, The PRISMA Group (2009). *P*referred *R*eporting *I*tems for *S*ystematic Reviews and *M*eta-*A*nalyses: The PRISMA Statement. PLoS Med 6(6): e1000097. doi:10.1371/journal.pmed1000097

For more information, visit www.prisma-statement.org.
